# The efficacy and safety of transcatheter aortic valve replacement in elderly patients with aortic stenosis

**DOI:** 10.4314/ahs.v25i1.20

**Published:** 2025-03

**Authors:** Jinghui An, Fengwu Shi, Huajun Wang, Hang Zhang, Su Liu

**Affiliations:** 1 Hebei Medical University, Shijiazhuang, China; 2 Department of Cardiac Surgery, the Second Hospital of Hebei Medical University, Shijiazhuang, China

**Keywords:** Valvular heart disease, Aortic stenosis, Elderly patients, Transcatheter aortic valve replacement, Echocardiography

## Abstract

**Background:**

Transcatheter aortic valve replacement (TAVR) is a promising treatment for aortic stenosis (AS). However, few clinical studies have concentrated on the application of TAVR in elderly AS patients. This study aimed to explore the efficacy and safety of TAVR in elderly patients with AS.

**Methods:**

Clinical and imaging data of 143 AS patients undergoing Transcatheter aortic valve replacement were retrospectively analyzed. These patients were divided into non-elderly group (< 65 years old) and elderly group (≥ 65 years old), and the baseline characteristics and clinical outcomes were compared between the two groups.

**Results:**

The baseline characteristics showed no significant difference between the two groups. After surgery, echocardiography revealed that the forward flow velocity of aortic valve was higher in the non-elderly group than that in the elderly group (234.2 ± 49.8 cm/s vs. 209.9 ± 57.7 cm/s, P=0.022), while there was no significant difference in other indicators. The operation time (197.4 ± 63.5 min vs. 194.2± 67.8 min, P=0.811), length of hospital stay (14.0±7.3 days vs.14.2±7.6 days, P=0.874), incidence of perioperative complications (2.2% vs. 4.1%, P=1.000), and mortality rate (0.0% vs. 1.0%, P=1.000) sho wed no significant differences between the two groups.

**Conclusion:**

Transcatheter aortic valve replacement is an effective and safe therapeutic approach for elderly patients with aortic stenosis.

## Introduction

The incidence of valvular heart disease is closely associated with age. Aortic stenosis (AS) is one of the most prevalent valve diseases, with a high incidence of up to 5% in elderly individuals^1^. An initial nationwide survey was conducted in China, including 8,929 individuals aged over 60 years old from 69 hospitals across 28 provinces and municipalities. The survey revealed that 5.1% of the elderly patients had aortic stenosis^1^. Another retrospective survey in China found that the prevalence of AS was 0.16% in inpatients younger than 65 years old, and it increased to 0.41% and 0.56% in those aged 65-74 and over 75 years old, respectively^2^. In a separate study conducted in China, it was discovered that 0.39% to 0.66% of outpatients aged over 65 years old, who underwent echocardiography, were diagnosed with severe AS^3^. With China's population entering an aging society, the burden of AS is increasing. A recent epidemiological survey in China highlighted degeneration as the leading cause of AS^4^. Surgical aortic valve replacement (SAVR) has long been the preferred treatment strategy for patients with symptomatic severe AS, as it can improve symptoms and increase survival rates. However, elderly AS patients have a high risk of comorbidities, making surgery risky. A study based on medical records revealed that a significant percentage of patients diagnosed with valvular heart disease, up to 30%, were deemed ineligible for surgery due to their elevated surgical risk^5^. Transcatheter aortic valve replacement (TAVR) is a minimally invasive valve replacement technique that was first performed in 2002. It does not require open-heart surgery or cardiopulmonary bypass^6^. Over time, as clinical evidence has accumulated and medical devices have improved, the indications for TAVR have expanded. It is now not only used for patients with high surgical risk or surgical contraindications but also for patients with intermediate or low-risk AS^7^,^8^. This means that TAVR has become a new treatment alternative for elderly AS patients. Despite this, only a few clinical studies have focused on the application of TAVR in elderly patients. Therefore, the aim of the present study is to explore the safety and efficacy of TAVR in elderly AS patients using accumulated data.

## Patients and Methods

### Patients

The clinical data of 143 AS patients who underwent TAVR in our hospital from August 2018 to June 2022 were retrospectively analyzed. There were 85 men and 58 women, with an average age of 67.9±6.6 years old. These patients were divided into non-elderly group (< 65 years old) and elderly group (≥ 65 years old based on their age. This study was approved by the Ethics Committee of the Second Hospital of Hebei Medical University. Prior to the commencement of the study, written informed consents were acquired from all participants. The inclusion criteria were as follows: a) patients with a definite diagnosis of AS; and b) patients who had undergone TAVR. The exclusion criteria were as follows: a) patients with contraindications for anticoagulation therapy; b) patients with acute cerebrovascular events; and c) patients with coronary heart disease requiring concurrent revascularization.

### Pre-operative preparation

All patients underwent preoperative transthoracic echocardiography and coronary multi-slice spiral computed tomography (MSCT). The diameter of the aortic annulus was measured by echocardiography in the short-axis view. The CT data were analyzed and reconstructed using the 3MENSIO software (Pie Medical Imaging, Amsterdam, Netherlands), along with the measurement on the aortic valve annulus plane, outflow tract plane, sinotubular junction plane, and ascending aorta plane, so as to assess the sizes of the left-, right-, and non-coronary sinuses and the coronary ostium height. A valve oappropriate size was selected according to the assessment results and the findings of intraoperative angiography.

### Surgical methods

Temporary endocardial pacing leads were implanted into the right ventricle through the internal jugular vein under monitored anesthesia. The size of the implanted stent-valve was determined according to the inner diameter of the aortic valve annulus, which was measured on preoperative CT. The stentvalve was assembled at the beginning of the procedure. According to the results of preoperative CT assessment of the lower extremity arteries, dissection was performed via the main approach, which enabled the puncture via the femoral artery under direct vision, and a 10F arterial sheath was placed. A 6-F pigtail catheter was placed into the bottom of the non-coronary sinus via the contralateral accessory approach, and aortic root angiography was performed to verify the projection angle. The bottom planes of three aortic sinuses were made completely perpendicular to the video plane, and these three sinuses were lined up from the left to the right in the order of non-coronary sinus, right coronary sinus, and left coronary sinus. A pigtail catheter was placed into the left ventricle via the main approach, and the Lunderquist exchange guide wire was used. In anticipation of the release of the valve, a sheath with a size of either 20F or 18F was utilized during preparation. The valve delivery device was inserted to the aortic root along the main approach, and the temporary pacemaker was adjusted to 120-180 beats/min during release. According to the structural characteristics of different roots, different release strategies were adopted to fully fit the stent-valve to the valve annulus. After completion of the release, the delivery system was withdrawn and the pigtail catheter was re-located into the left ventricle. Aortic root angiography was performed through the accessory approach to verify the position and function of the stent-valve. The Venus-A valve (VenusMedTech, Hangzhou, China) was utilized in the procedure, and the valve models 23, 26, 29, and 32 were selected.

### Statistical analysis

Statistical analysis was performed using the SPSS 19.0 software (IBM, Armonk, NY, USA). Normally distributed quantitative data were presented as the mean ± standard deviation, and abnormally distributed quantitative data were expressed as the median and interquartile range. The t-test was used to compare the normally distributed measurement data, and the rank-sum test was utilized to compare abnormally distributed quantitative data. Count data are expressed as frequency (percentage). They were compared using the *χ*^2^ test or the Fisher's exact test (when T<5). P<0.05 was considered statistically significant.

## Results

### General data

A total of 143 patients aged 53-84 years old were included in the study. Among them, there were 45 (31.5%) patients in the non-elderly group (aged <65 years old) and 98 patients (68.5%) in the elderly group (aged ≥ 65 years old). There were no significant differences in gender, body mass index (BMI), smoking history, incidence of hypertension, incidence of diabetes, incidence of hyperlipidemia, incidence of coronary heart disease, incidence of arrhythmia, incidence of peripheral arterial diseases, incidence of previous myocardial infarction, and incidence of previous stroke between the two groups (all P>0.05) (Table 1). The preoperative New York Heart Association (NYHA) grading, the left ventricular ejection fraction (LVEF), left ventricular end-diastolic diameter, moderate/higher degrees of mitral regurgitation, tricuspid regurgitation and aortic valve regurgitation, and aortic valve forward pressure difference were assessed by transthoracic echocardiography, which also showed no significant difference (all P>0.05) (Table 1).

**Table 1:** Primer sequences for RT-PCR

Gene name	Forward (5′>3′)	Reverse (5′>3′)
Collagen I	GAGGGCCAAGACGAAGACATC	CAGATCACGTCATCGCACAAC
Collagen II	TGGACGATCAGGCGAAACC	GCTGCGGATGCTCTCAATCT
Collagen III	ATGTTGTGCAGTTTGCCCAC	TCGTCCGGGTCTACCTGATT
Aggrecan	GGTGAACCAGTTGTGTTGTC	CCGTCCTTTCCAGCAGTC
CTGF	GAGCGGAGAGTCCTTCCAGAG	GGCCAAATGTGTCTTCCAGTC
VEGF-A	CTTGCCTTGCTGCTCTACC	CACACAGGATGGCTTGAAG
GAPDH	ACAACTTTGGTATCGTGGAAGG	GCCATCACGCCACAGTTTC

RT-PCR, quantitative reverse-transcription polymerase chain reaction

### Postoperative transthoracic echocardiography

Postoperative transthoracic echocardiography revealed that the LVEF, left ventricular end-diastolic diameter, moderate/higher degrees of mitral regurgitation, tricuspid regurgitation and aortic valve regurgitation, aortic valve forward pressure, and paravalvular regurgitation were not significantly different between the two groups (all P>0.05) (Table 2). However, it was found that the forward flow velocity of aortic valve was higher in the non-elderly group than that in the elderly group (234.2±49.8 cm/s vs. 209.9±57.7 cm/s, P=0.022).

**Table 2:** Primer sequences of the siRNA-null and –CTGF

Gene name	Forward (5′>3′)	Reverse (5′>3′)
null	UUCUCCGAACGUGUCACGUTT	ACGUGACACGUUCGGAGAATT
CTGF-1	CCAGACCCAACUAUGAUUATT	UAAUCAUAGUUGGGUCUGGTT
CTGF-2	CCAAGCCUAUCAAGUUUGATT	UCAAACUUGAUAGGCUUGGTT
CTGF-3	GCUAAAUUCUGUGGAGUAUTT	AUACUCCACAGAAUUUAGCTT

### Follow-up outcomes

The operation time (197.4±63.5 min vs. 194.2±67.8 min, P=0.811) and length of hospital stay (14.0±7.3 days vs. 14.2 ± 7.6 days, P=0.874) showed no significant differences. During the perioperative period, 1 patient in the non-elderly group developed transient third-degree atrioventricular block and complete left bundle branch block, in whom no permanent pacemaker was implanted; 4 patients in the elderly group developed complications, including complete left bundle branch block (n=2), transient third-degree atrioventricular block (n=1), and pericardial effusion (n=1); the condition was improved after incision and drainage. There was no significant difference in the incidence of perioperative complications between the two groups (2.2% vs. 4.1%, P=1.000). During the follow-up period, 1 patient in the elderly group died of iliac vessel rupture, and there was no significant difference in the overall mortality rate between the two groups (0.0% vs. 1.0%, P=1.000) (Table 2).

## Discussion

Aortic stenosis (AS) is a common heart valve disease among the elderly. In recent years, significant progress has been made in transcatheter aortic valve replacement (TAVR), and large-scale clinical studies such as PARTNER^9^ have demonstrated its advantages in reducing postoperative complications and improving prognosis. Specifically, for patients with cardiac insufficiency, TAVR has shown to improve left ventricular ejection fraction (LVEF) within one month after the procedure, leading to a significant reduction in long-term mortality^10^. The present study confirms that elderly AS patients who undergo TAVR have a comparable prognosis to non-elderly patients. Other studies have also shown that TAVR is a safe and effective treatment for AS patients aged 80 years and older^11^,^12^.

The 2020 American College of Cardiology (ACC) and American Heart Association (AHA) guidelines for the Management of Patients with Valvular Heart Disease, and the 2021 European Society of Cardiology (ESC) and European Society for Cardiothoracic Surgery (EACTS) guidelines for the management of valvular heart disease, have updated the recommendations for the management of AS patients undergoing TAVR^13^,^14^. These new guidelines emphasize individualization, refinement, and specialization in decision-making for surgical treatment options, taking into account factors such as patient age/life expectancy, frailty, prosthetic valve durability, anatomical variations, cardiac and non-cardiac factors (e.g., lung, liver, and kidney conditions), surgical risk, and patient preferences^15^.

Currently, TAVR is increasingly offered as an alternative to surgical aortic valve replacement (SAVR) for patients with symptomatic severe AS and those with intermediate or higher surgical risk. It has been shown to have comparable outcomes to SAVR^16^-^20^. Additionally, the outcome of patients with low ejection fraction (EF) severe AS following TAVR is as good as that of patients with preserved EF^21^. Symptomatic improvement, as reflected by changes in the New York Heart Association (NYHA) functional class, has been observed in several studies. TAVR has also been found to improve symptoms of heart failure, quality of life, neurohormonal activation, and reverse cardiac remodeling^22^,^23^. TAVR has revolutionized the management of AS patients, demonstrating favorable hemodynamic changes compared to SAVR^23^. Studies examining the effect of baseline EF on TAVR outcomes have yielded divergent and inconsistent results. However, studies have shown that systolic dysfunction (LVEF < 30%) is not significantly associated with higher mortality rates in patients undergoing TAVR^24^,^25^.

Elderly AS patients are a major clinical concern due to their advanced age, frailty, low exercise capacity, and multiple comorbidities such as renal insufficiency, diabetes, cerebrovascular diseases, and chronic obstructive pulmonary disease^26^,^27^. Despite the proven safety and efficacy of TAVR in the elderly population, these patients often have low levels of physical activity and significantly reduced quality of life^28^. Cardiac rehabilitation, which encompasses physical, psychological, and social management services, plays a crucial role in improving exercise capacity, quality of life, psychological well-being, frailty, and short-term survival, particularly in elderly AS patients^29^. Cardiac rehabilitation has been recommended as a class I intervention for the prevention and treatment of cardiovascular diseases in several countries.

Recent studies have also shown that cardiac magnetic resonance (CMR) and other imaging modalities can comprehensively assess myocardial remodeling after TAVR, further optimizing rehabilitation management^30^. The present study has limitations, including its small-scale and single-center design, and the lack of long-term follow-up data. Theefore, further large-scale, multicenter studies with long-term follow-up data are needed to confirm the results of this study and address these limitations.

## Conclusion

TAVR has demonstrated comparable outcomes in elderly AS patients, making it an effective and safe therapeutic strategy for this population.

## Figures and Tables

**Figure 1:**
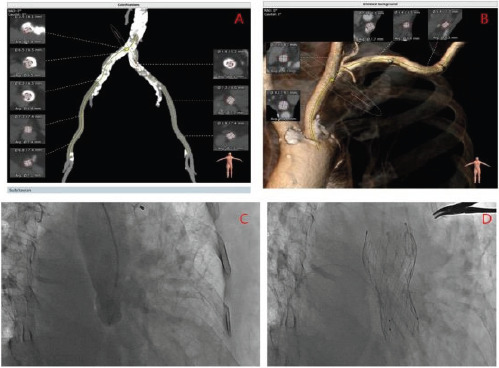
An old AS patient undergoing TAVR. (A) Severe calcification of the iliac artery; (B) Transcarotid approach; (C) Aortic root angiography; (D) Release of Venus-A valve

## References

[1] Xu H, Liu Q, Cao K (2022). Distribution, Characteristics, and Management of Older Patients with Valvular Heart Disease in China: China-DVD Study. JACC Asia.

[2] Hu P, Liu XB, Liang J, Zhu QF, Pu CX, Tang MY (2017). A hospital-based survey of patients with severe valvular heart disease in China. Int J Cardiol.

[3] Pan W, Zhou D, Cheng L, Shu X, Ge J (2013). Candidates for transcatheter aortic valve implantation may be fewer in China. Int J Cardiol.

[4] Nkomo VT, Gardin JM, Skelton TN, Gottdiener JS, Scott CG, Enriquez-Sarano M (2006). Burden of valvular heart diseases: a population-based study. Lancet.

[5] (2021). Report on Cardiovascular Health and Diseases in China 2020 expert panel. Key points of Report on Cardiovascular Health and Diseases in China 2020. Chinese Journal of Cardiovascular Research.

[6] Iung B, Baron G, Butchart EG, Delahaye F, Gohlke-Barwolf C, Levang OW (2003). A prospective survey of patients with valvular heart disease in Europe: The Euro Heart Survey on Valvular Heart Disease. Eur Heart J.

[7] Cribier A, Eltchaninoff H, Bash A, Borenstein N, Tron C, Bauer F (2002). Percutaneous transcatheter implantation of an aortic valve prosthesis for calcific aortic stenosis: first human case description. Circulation.

[8] Mack MJ, Leon MB, Thourani VH, Makkar R, Kodali SK, Russo M (2019). Transcatheter Aortic-Valve Replacement with a Balloon-Expandable Valve in Low-Risk Patients. New Engl J Med.

[9] Popma JJ, Deeb GM, Yakubov SJ, Mumtaz M, Gada H, O'Hair D (2019). Transcatheter Aortic-Valve Replacement with a Self-Expanding Valve in Low-Risk Patients. New Engl J Med.

[10] Leon MB, Smith CR, Mack M, Miller DC, Moses JW, Svensson LG (2010). Transcatheter aortic-valve implantation for aortic stenosis in patients who cannot undergo surgery. New Engl J Med.

[11] Kolte D, Bhardwaj B, Lu M, Alu MC, Passeri JJ, Inglessis I (2022). Association Between Early Left Ventricular Ejection Fraction Improvement After Transcatheter Aortic Valve Replacement and 5-Year Clinical Outcomes. JAMA Cardiol.

[12] Xu DH, Luo XJ, Zhang J (2021). Application of transcatheter aortic valve replacement in very old patients with aortic stenosis. Chinese Journal of Multiple Organ Diseases in the Elderly.

[13] Oh S, Kim JH, Hwang CH, Hyun DY, Cho KH, Kim MC (2022). Comparison of outcomes after transcatheter aortic valve replacement between elderly (65-79 years) and super-elderly (>/=80 years) patients. Medicine.

[14] Otto CM, Nishimura RA, Bonow RO, Carabello BA, Erwin JR, Gentile F (2021). 2020 ACC/AHA Guideline for the Management of Patients With Valvular Heart Disease: Executive Summary: A Report of the American College of Cardiology/American Heart Association Joint Committee on Clinical Practice Guidelines. Circulation.

[15] Vahanian A, Beyersdorf F, Praz F, Milojevic M, Baldus S, Bauersachs J (2022). 2021 ESC/EACTS Guidelines for the management of valvular heart disease. Eur Heart J.

[16] Leon MB, Smith CR, Mack MJ, Makkar RR, Svensson LG, Kodali SK, Thourani VH, Tuzcu EM, Miller DC, Herrmann HC (2016). Transcatheter or surgical aortic-valve replacement in intermediate-risk patients. N Engl J Med.

[17] Nishimura RA, Otto CM, Bonow RO, Carabello BA, Erwin JP, Fleisher LA, Jneid H, Mack MJ, McLeod CJ, O'Gara PT (2017). 2017 AHA/ACC focused update of the 2014 AHA/ACC guideline for the management of patients with valvular heart disease: a report of the American College of Cardiology/American Heart Association Task Force on Clinical Practice Guidelines. Circulation.

[18] Kodali SK, Williams MR, Smith CR, Svensson LG, Webb JG, Makkar RR, Fontana GP, Dewey TM, Thourani VH, Pichard AD (2012). Two-year outcomes after transcatheter or surgical aortic-valve replacement. N Engl J Med.

[19] Mack MJ, Leon MB, Smith CR, Miller DC, Moses JW, Tuzcu EM, Webb JG, Douglas PS, Anderson WN, Blackstone EH (2015). 5-year outcomes of transcatheter aortic valve replacement or surgical aortic valve replacement for high surgical risk patients with aortic stenosis (PARTNER 1): a randomised controlled trial. Lancet.

[20] Heidenreich PA, Bozkurt B, Aguilar D, Allen LA, Byun JJ, Colvin MM, Deswal A, Drazner MH, Dunlay SM, Evers LR (2022). 2022 AHA/ACC/HFSA guideline for the management of heart failure: a report of the American College of Cardiology/American Heart Association Joint Committee on Clinical Practice Guidelines. J Am Coll Cardiol.

[21] Ternacle J, Faroux L, Alperi A, Muntané-Carol G, Delarochellière R, Paradis JM, Kalavrouziotis D, Mohammadi S, Dumont E, Beaudoin J (2021). Impact of left-ventricular dysfunction in patients with high- and low- gradient severe aortic stenosis following transcatheter aortic valve replacement. The Can J Cardiol.

[22] Dong M, Wang L, Tse G (2023). Effectiveness and safety of transcatheter aortic valve replacement in elderly people with severe aortic stenosis with different types of heart failure. BMC Cardiovasc Disord.

[23] Clavel MA, Webb JG, Rodés-Cabau J, Masson JB, Dumont E, De Larochellière R, Doyle D, Bergeron S, Baumgartner H, Burwash IG (2010). Comparison between transcatheter and surgical prosthetic valve implantation in patients with severe aortic stenosis and reduced left ventricular ejection fraction. Circulation.

[24] Baron SJ, Arnold SV, Herrmann HC, Holmes DR, Szeto WY, Allen KB, Chhatriwalla AK, Vemulapali S, O'Brien S, Dai D (2016). Impact of ejection fraction and aortic valve gradient on outcomes of transcatheter aortic valve replacement. J Am Coll Cardiol.

[25] Elmariah S, Palacios IF, McAndrew T, Hueter I, Inglessis I, Baker JN, Kodali S, Leon MB, Svensson L, Pibarot P (2013). Outcomes of transcatheter and surgical aortic valve replacement in high-risk patients with aortic stenosis and left ventricular dysfunction: results from the Placement of Aortic Transcatheter Valves (PARTNER) trial (cohort A). Circ Cardiovasc Interv.

[26] Liu QR, Chen Y, Xu HY (2022). New progress in transcatheter aortic valve replacement in elderly patients with aortic stenosis. Chinese Journal of Medicine.

[27] Xie MR, Xu HY, Wu YJ (2022). Research Advances in cardiac rehabilitation in patients undergoing transcatheter aortic valve replacement. Chinese Circulation Journal.

[28] Pelliccia A, Sharma S, Gati S, Back M, Borjesson M, Caselli S (2021). 2020 ESC Guidelines on sports cardiology and exercise in patients with cardiovascular disease. Eur Heart J.

[29] Ambrosetti M, Abreu A, Corra U, Davos CH, Hansen D, Frederix I (2020). Secondary prevention through comprehensive cardiovascular rehabilitation: From knowledge to implementation. 2020 update. A position paper from the Secondary Prevention and Rehabilitation Section of the European Association of Preventive Cardiology. Eur J Prev Cardiol.

[30] Lange T, Backhaus SJ, Beuthner BE, Topci R, Rigorth KR, Kowallick JT (2022). Functional and structural reverse myocardial remodeling following transcatheter aortic valve replacement: a prospective cardiovascular magnetic resonance study. J Cardiovasc Magn R.

